# Host records and geographical distribution of *Corynosoma magdaleni*, *C. semerme* and *C. strumosum* (Acanthocephala: Polymorphidae)

**DOI:** 10.3897/BDJ.8.e50500

**Published:** 2020-04-06

**Authors:** Sonja Leidenberger, Sven Boström, Matthew Thomas Wayland

**Affiliations:** 1 School of Bioscience, Department of Biology and Bioinformatics, University of Skövde, Skövde, Sweden School of Bioscience, Department of Biology and Bioinformatics, University of Skövde Skövde Sweden; 2 Swedish Museum of Natural History, Department of Zoology, Stockholm, Sweden Swedish Museum of Natural History, Department of Zoology Stockholm Sweden; 3 University of Cambridge, Cambridge, United Kingdom University of Cambridge Cambridge United Kingdom

**Keywords:** Acanthocephala, *
Corynosoma
*, host record, life cycle, Northern Hemisphere, seal and zoogeography

## Abstract

A literature survey was conducted to investigate the host and geographical distribution patterns of three *Corynosoma* species (Acanthocephala: Polymorphidae), *viz. C. magdaleni*, *C. semerme* and *C. strumosum*. All three species appear to be restricted to the Northern Hemisphere. Occurrence records of *C. magdaleni* are limited to the Northern Atlantic coasts, while *C. semerme* has a circumpolar distribution. The geographical range of *Corynosoma strumosum* encompasses the distributions of the other two species, but also extends into warmer southern regions. Some *Corynosoma* populations are living with their definitive hosts in very isolated locations, such as in the brackish Baltic Sea or different freshwater lakes (e.g. Lake Saimaa). All three species have a heteroxenous life cycle, comprising a peracaridan intermediate host, a fish paratenic host and a mammalian definitive host. Occasionally, an acanthocephalan may enter an accidental host, from which it is unable to complete its life cycle. The host records reported here are categorised by type, *i.e.* intermediate, paratenic, definitive or accidental. While most of the definitive hosts are shared amongst the three *Corynosoma* species, *C. strumosum* showed the broadest range of paratenic hosts, which reflects its more extensive geographical distribution. One aim of this study and extensive literature summary is to guide future sampling efforts and therewith contribute to throw more light on the on-going species and morphotype discussion for this interesting parasite species.

## Introduction

The genus *Corynosoma* comprises 43 marine species (Amin 2013), that infect mammals and piscivorous birds. Aznar et al. (2006) showed that the original concept of *Corynosoma* was not a monophyletic genus. Phylogenetic analysis revealed that the marine *Corynosoma* species were more closely related to the genus *Andracantha* Schmidt, 1975 than to the species living in freshwater habitats. Aznar et al. (2006) erected the genus *Pseudocorynosoma* Aznar, Pérez-Ponce de León and Raga, 2006 for the freshwater species. Recent phylogenetic analysis based on ribosomal ITS1-5.8S-ITS2 and COI sequences showed that *Corynosoma* spp., hosted by Pinnipedia and marine Mustelidae formed a monophyletic group (Waindok et al. 2018).

The systematics of *Corynosoma* species from seals in northern Europe have recently been investigated using both molecular markers (Waindok et al. 2018) and morphology (Leidenberger et al. 2019). These studies have highlighted the limitations of using traditional morphological characters alone for diagnosing species. To determine the true species diversity in this genus, tandem morphological and molecular studies will be required. To guide future sampling efforts, we have conducted a comprehensive literature survey of the host and geographical distribution of three commonly encountered taxa: *C. magdaleni* Montreuil, 1958, *C. semerme* (Forssell, 1904) Lühe, 1911 and *C. strumosum* (Rudolphi, 1802) Lühe, 1904.

## Material and methods

### Host and geographical records

Host and geographical records for the three acanthocephalan taxa were collected from literature. Manual searches were conducted using Pubmed, Web of Science and Google Scholar. Additionally, we used the R (R Core Team 2019) package helminthR (Dallas 2016) to extract records from the Host-Parasite Database of The Natural History Museum in London (Gibson et al. 2005). Geographical records for each *Corynosoma* species were summarised by MEOW ecoregions (Spalding et al. 2007) and then plotted as a distribution map using the meow R-package (Byrnes 2016).

### Nomenclature

For species names, we followed the nomenclature given by the Catalogue of Life (2019 Annual Checklist: Roskov et al. 2019) and WoRMS (WoRMS Editorial Board 2019).

The term *accidental host* was used here for a host, where the parasite usually is not found, because the host is not suitable for the parasite's development. This can mean that the accidental host also becomes a dead-end, because the life cycle of the parasite is blocked (e.g. no observed gravid female and/or mature individuals) and the parasite does not reach its definitive host. Since in many literature references, this detailed information was not given, we used the term accidental host as generic term for all of the dead-end/accidental/incidental host records we found.

## Results and discussion

### Zoogeography

**Figure 1.**
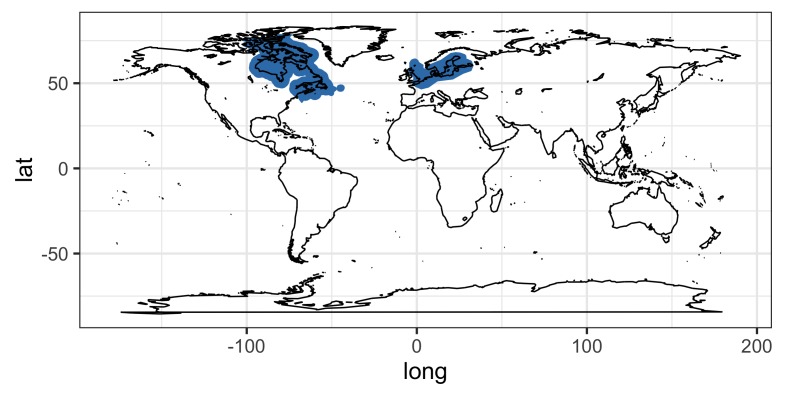
Geographical distribution of *Corynosoma magdaleni*.

**Figure 2.**
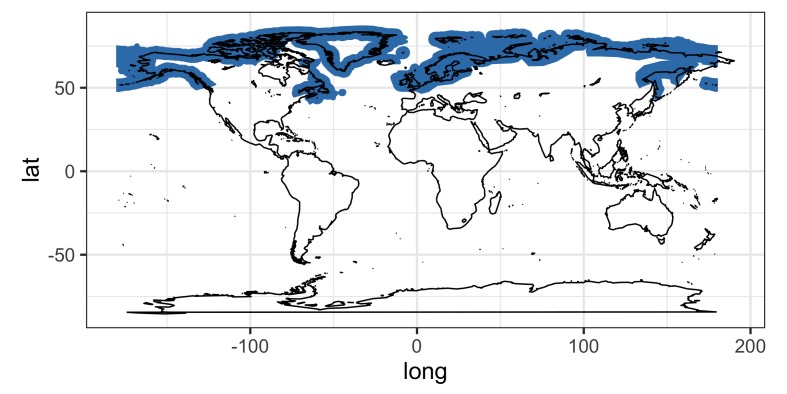
Geographical distribution of *Corynosoma semerme*.

**Figure 3.**
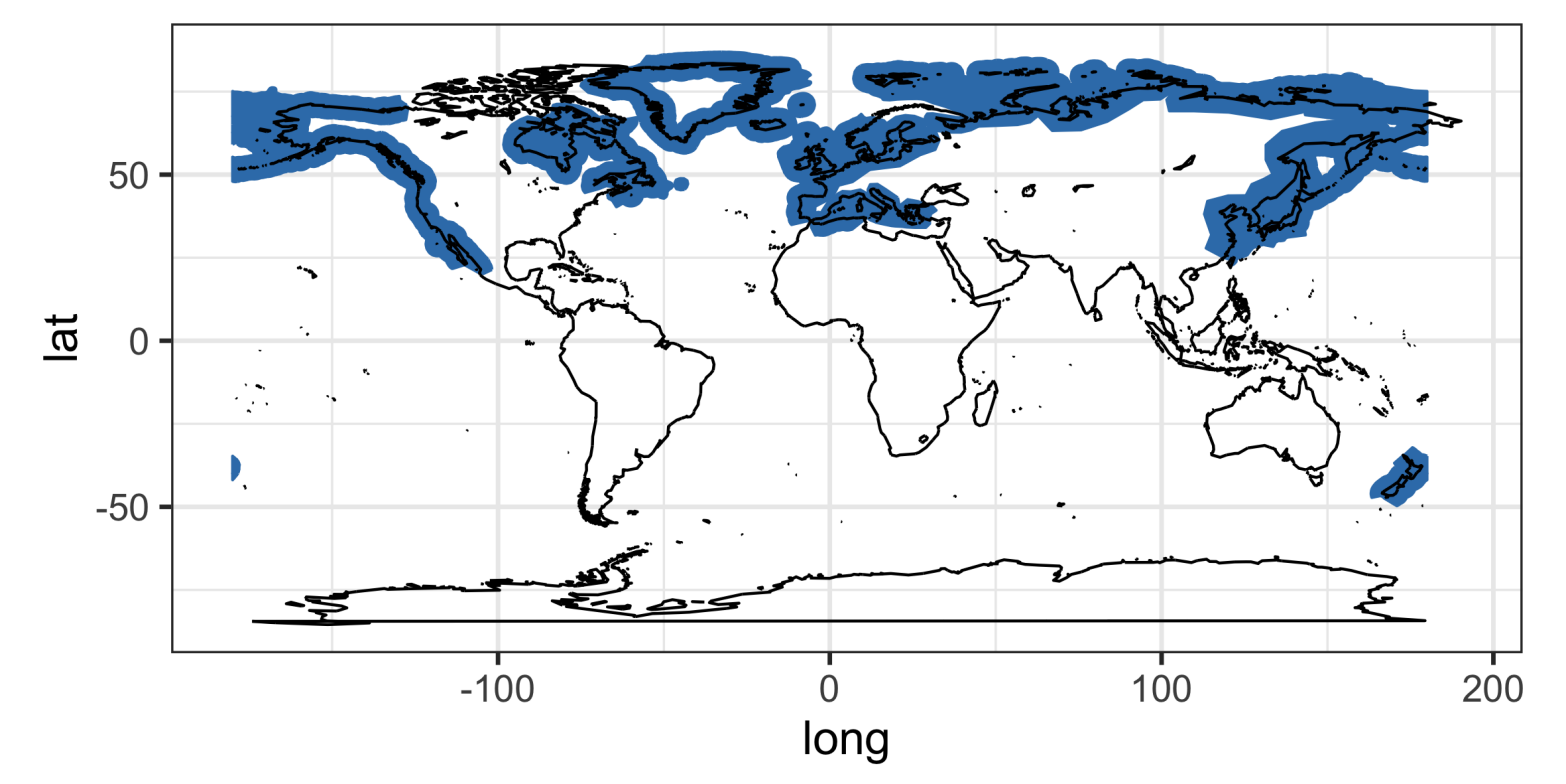
Geographical distribution of *Corynosoma strumosum*.

Our literature survey on the zoogeography showed that the three species of *Corynosoma* (*C. magdaleni*, *C. semerme* and *C. strumosum*) and their definitive hosts are restricted to the Northern Hemisphere (Figs 1, 2, 3). The relatively few records for *C. magdaleni* suggest that this taxon is restricted to the North Sea, Baltic Sea and northwest Atlantic. By contrast, *C. semerme* has a circumpolar distribution, having been reported from the Arctic Ocean, north Atlantic and north Pacific (e.g. Popov and Fortunato 1987, Kaimoto et al. 2018, Waindok et al. 2018) (Fig. 2). The geographical range of *C. strumosum* encompasses the distributions of the other two taxa, but also extends into more southern regions, such as the Mediterranean Sea and coast of California (Fig. 3).

In most of the Baltic Sea studies, *C. semerme* was the species most commonly found in seals, followed by *C. magdaleni* and only rare infections were found for *C. strumosum* (Nickol et al. 2002, Sinisalo and Valtonen 2003, Leidenberger and Bäcklin 2008, Waindok et al. 2018). Co-infections have been observed (Nickol et al. 2002, Leidenberger and Bäcklin 2008, Leidenberger et al. 2019), most commonly involving *C. semerme* and *C. magdaleni* (Sinisalo and Valtonen 2003, Leidenberger and Bäcklin 2008, Waindok et al. 2018). Interestingly, land-locked seal species show no mixed infections, but only single infections, for example, *Phoca hispida saimensis* in Lake Saimaa only by *C. magdaleni* (Sinisalo and Valtonen 2003) and *Pusa caspica* in the Caspian Sea only by *C. strumosum* (Amin et al. 2011). García-Varela et al. (2005) concluded that the *Corynosoma* species found in the Caspian Sea is *C. caspicum* and not *C. strumosum*. Indeed, *C. strumosum* from the land-locked Caspian seal recorded by Amin et al. 2011 was characterised with proboscis hooks and trunk spines distinct from other Northern European morphological descriptions (Nickol et al. 2002, Waindok et al. 2018, Leidenberger et al. 2019), suggesting that they may not be conspecific. Surprisingly, *Corynosoma caspicum* Golvan and Mokhayer, 1973, described based on immature specimens from sturgeons in Caspian Sea (Golvan and Mokhayer 1973) and often found in three-spined stickleback (*Gasterosteus aculeatus*) (Niksirat et al. 2006), has not yet been reported in *Pusa caspica* or another definitive host. Waindok et al. (2018) described some individuals of *C. strumosum* from the German North and Baltic Seas with different proboscis morphology, similar to those described by Amin et al. (2011) and stated this as *C. magaleni* isolate Pv1NS instead. Additionally, they found a cryptic species refered to as "Candidatus *Corynosoma nortmeri* sp. nov."' Waindok et al. (2018) in the European study area.

The extent of the geographical and host ranges of the three taxa correlates with the year of their description. *Corynosoma strumosum* was the first species of the genus to be described (Rudolphi 1802) and has, by far, the broadest geographical and host distributions. *Corynosoma semerme*, described by Forssell (1904), appears to have a slightly more limited geographical range and far fewer paratenic host records. Relatively few hosts have been reported for *C. magdaleni*, described by Montreuil (1958) and its geographical range appears to be restricted to the northern Atlantic, in contrast to the circumpolar distributions of the other two species. Up until its description in 1958, specimens of *C. magdaleni* were almost certainly assigned to the morphologically similar *C. strumosum.* The potential bias resulting from taxon age should be kept in mind when comparing the reported host and geographical distributions of the three taxa (Fig. 3).

### Overview of the life cycle

The heteroxenous life cycle of *Corynosoma* species involves a peracaridan intermediate host, a paratenic host (fish) and a mammalian definitive host. While there are numerous studies on *C. semerme* and *C. strumosum* (Tables 3, 4, 5, 6, 7, 8), there have been few studies on *C. magdaleni*, besides its original description by Montreuil 1958 (Tables 1, 2). The intermediate host of *C. magdaleni* is unknown. Lake Saimaa may be a good location to search for the intermediate host of *C. magdaleni*, because the other *Corynosoma* species appear to be absent from this land-locked waterbody (Sinisalo and Valtonen 2003, Sinisalo et al. 2003). A total of five species of paratenic host (all Actinopterygii) have been reported for *C. magdaleni*; three from Canada and two from the Baltic Sea (Table 1). In Canada, only *C. magdaleni* is reported together with *C. wegeneri* Heinze, 1934. In the definitive hosts (Table 2), the species seems to prefer the last part of the small intestine (ileum) and the colon. *Corynosoma semerme* has been reported from the whole intestine, but is typically found at a higher density in the large intestine, especially the cecum and rectum (Nickol et al. 2002, Leidenberger and Bäcklin 2008), where *C. strumosum* and *C. magdaleni* are not generally observed. Microhabitat segregation might facilitate reproductive isolation of the three species, especially in mixed infections that are often observed in seals from the Baltic Sea (Nickol et al. 2002, Sinisalo and Valtonen 2003, Leidenberger and Bäcklin 2008), but less commonly in the North Sea (Waindok et al. 2018). While the three *Corynosoma* species share most of their definitive hosts, they show some differences in their paratenic hosts. We found that *C. strumosum* shares three paratenic hosts with both other species, none with *C. magdaleni* only, but > 20 paratenic hosts are shared with *C. semerme.* More than 80 paratenic hosts were described for *C. strumosum* and these are, up to now, not reported from any of the other two species (Tables 1, 4, 6).

While for *C. magdaleni*, all definitive host records belong to the family Phocidae (order Carnivora), the definitive hosts for *C. semerme* include, besides numerous Phocidae, also Otariidae and Odobenidae from the same order and Monodontidae (belonging to the order Artiodactyla) (Table 3). For this reason, more paratenic hosts of *Corynosoma semerme* in Europe and North America are reported (Table 4), but which all are also Actinopterygii. *Monoporeia affinis* (Amphipoda) is the only known intermediate host of *Corynosoma semerme* (Nybelin 1924). Some further intermediate hosts are suggested for the Baltic Sea area, mainly isopods (*Saduria entomon*, *Asellus aquaticus*), amphipods (*Gammarus* spp.) ostracods and Mysidae, because such invertebrate species were found in the intestines of infected paratenic fish (e.g. Valtonen and Julkunen 1995). From North America, the intermediate host is still unknown.

For *C. strumosum*, an arthropod (probably an amphipod) is suggested as an intermediate host in North America (Van Cleave 1953), which is similar to the suggested intermediate hosts in Europe. Recently, Skorobrechova and Nikishin (2019) found corynosome cystacanths in *Spinulogammarus ochotensis* (Brandt, 1851), which further confirmed amphipods as potential intermediate hosts. *Corynosoma strumosum* seems to have fewer definitive hosts than *C. semerme* (Table 5), but the species shows also numerous reported paratenic hosts in Europe, North America, the Caspian Sea, Sea of Okhotsk and Northwest Pacific (Table 6), belonging to the classes Actinoptergyii and Petromyzonti. Even if the *Corynosoma* spp. are observed in many fish species, they are not able to become sexually mature in fish (Forssell 1905, Lundström 1942). This is the reason why fish may only play an important role as paratenic host.

The definitive host of *Corynosoma* has fish as prey, but numerous fish-eating species become accidentally infected and become dead-end hosts (see Tables 7, 8 *C. semerme* and *C. strumosum*, respectively). While it is not known how long a life cycle of *Corynosoma* spp. takes, the development of the cystacanths to become mature helminths in seals is suggested to take 2-3 weeks (Helle and Valtonen 1981) and the season and/or temperature might have a strong effect on the sex-age structure of *Corynosoma* spp. (Helle and Valtonen 1981, Popov and Fortunato 1987).

Aznar et al. (2006) characterised the marine *Corynosoma* clade as cosmopolitan and, in the past, the marine genus was able to adapt their complex life cycle to extreme environments like the brackish Baltic Sea, Lake Saimaa and the Caspian Sea. It is also postulated that the right intermediate and paratenic host is available and serves as a reservoir for the cystacanth. Marine glacial relict species serve or are supposed to serve as intermediate hosts for *Corynosoma* species in the Baltic Sea (e.g. the amphipod *Monoporeia affinis* (Lindström, 1855) and the isopod *Saduria entomon* (Linnaeus, 1758)). Lundström (1942) discussed the idea that the *Corynosoma* spp., like *Echinorhynchus salmonis* Müller, 1784, may be a marine glacial relict species, because these species are also found in the Arctic Sea, White Sea and numerous freshwater and relict lakes (e.g. Lake Saimaa, Lake Ladoga, Lake Onega).

Sinisalo and Valtonen (2003) mentioned, that the *C. semerme* cystacanths, observed in Baltic fish, differed clearly in the morphological characters (trunk length), while *C. magdaleni* and *C. strumosum* cystacanths were hard to separate (*C. strumosum* only slightly larger than *C. magdaleni*). This is interesting, especially since also Waindok et al. (2018) mentioned some difficulties with the identification of *C. strumosum*. Acanthocephalans from harbour seals of the North Sea were initially diagnosed as *C. strumosum*, based on morphological characters; however, molecular markers (COI and ITS) indicated that they should have been assigned to *C. magdaleni*.

Another study, Hernández-Orts et al. (2017), based on partial sequences of the mitochondrial cytochrome *c* oxidase 1 gene (*cox1*), clearly segregated the Northern Hemisphere species (*C. magdaleni* and *C. strumosum*) and Southern Hemisphere species (*C. hannae* and *C. australe* Johnston, 1937). *Corynosoma hannae* from the Southern Hemisphere is most similar to *C. semerme* from the Northern Hemisphere, having no genital spines in females. It could be possible that parallel evolution may have evolved two similar complex parasitic *Corynosoma* systems in pinnipeds. It is still unresolved, whether the only report of *C. semerme* from the Southern Hemisphere by Johnston and Edmonds (1953) is *C. hannae* or not (discussed by Hernández-Orts et al. 2017).

Another possible assumption could be that the marine genus, *Corynosoma*, entered the Baltic Sea at the same time as their definitive hosts and this reflects their population history. The Baltic grey seal population is suggested to have diverged from the Eastern Atlantic/North Sea ones between 4,200 and 10,000 years ago (Fietz et al. 2016, Klimova et al. 2014), when the breeding habitats were shifted more easterly during the Baltic Sea formation. For the ringed seal, the allele frequency differentiation between the Baltic and Arctic populations were weak (Palo et al. 2001), while a northern invasion from continental seals during the Plio-Pleistocene to the basins was suggested to be most likely (Palo and Väinölä 2006). The ringed seal is the exclusive seal species in the land-locked Lake Saimaa, Finland. It also formed isolated populations in the Arctic and North-western Pacific (Popov and Fortunato 1987). The Caspian seal is another very isolated population and is supposed to be a relict species as well. Today, the ringed seal is very isolated in the Baltic Sea, as well as a small population of harbour seals (Härkönen et al. 2005).

## Conclusion

Obviously, with the geographical isolation, *Corynosoma* species show plastic morphological characters and possible morphotypes (e.g. Popov and Fortunato 1987, Amin et al. 2011). Which role the intermediate and paratenic host play in the isolation is not known today. Sinisalo and Valtonen (1998) found an indication of segregation of *C. magdaleni* and *C. strumosum* in their paratenic hosts in the Gulf of Bothnia, Baltic Sea, Finland. In general, there is too little information available on the ecology and distribution of each of the *Corynosoma* spp. Future studies should try to combine genetic and new morphological tools (like SEM and the proboscis profiler) to throw more light on the on-going species and morphotype discussion for *Corynosoma* spp. We see the greatest challenge is the access to good and sufficient material for further analyses. Our reported geographical distribution patterns (Suppl. material 1) and summary of definitive and paratenic hosts may contribute to and motivate further investigations on this interesting parasite group.

*Corynosoma semerme* and *C. strumosum* have extensive host and geographical ranges, providing opportunities for reproductive isolation of lineages. Speciation in acanthocephalans is often cryptic and Waindok et al. (2018) have already demonstrated the presence of a cryptic species, "Candidatus *Corynosoma nortmeri*," in the North Sea. Further work to investigate the species diversity in *Corynosoma* will require a tandem morphological and molecular study of acanthocephalans collected from all known hosts, throughout their geographical ranges. The present study should prove to be a useful guide for future sampling efforts.

## Supplementary Material

3CD18592-24ED-51A6-B7AB-F0EC796724FD 10.3897/BDJ.8.e50500.suppl1 Supplementary material 1 R script for generating geographical distribution maps. Data type R script File: oo_317281.R https://binary.pensoft.net/file/317281 Leidenberger S., Boström S. & Wayland M.

## Figures and Tables

**Table 1.** Paratenic host records for *Corynosoma magdaleni*. All Actinopterygii.

**Host order**	**Host family**	**Host species**	**Host vernacular name**	**Geographical locality**	**Reference(s)**
Perciformes	Percidae	*Gymnocephalus cernua* (Linnaeus, 1758) (as *Gymnocephalus cernuus*)	ruffe	Baltic Sea, North-eastern Bothnian Bay	Valtonen et al. 2001
Pleuronectiformes	Pleuronectidae	*Hippoglossus hippoglossus* (Linnaeus, 1758)	Atlantic halibut	Atlantic, Canada	Arai 1989
Pleuronectiformes	Pleuronectidae	*Hippoglossus hippoglossus* (Linnaeus, 1758)	Atlantic halibut	Magdalen Islands region of the Gulf of St. Lawrence	Montreuil 1958
Salmoniformes	Salmonidae	*Salvelinus fontinalis* (Mitchill, 1814)	brook charr	Tabusintac River, New Brunswick	Frimeth 1987
Scorpaeniformes	Cottidae	*Myoxocephalus quadricornis* (Linnaeus, 1758)	fourhorn sculpin	Baltic Sea, Bothnian Bay	Sinisalo and Valtonen 2003
Scorpaeniformes	Cottidae	*Myoxocephalus scorpius* (Linnaeus, 1758)	shorthorn sculpin	Atlantic, Canada	Arai 1989
Scorpaeniformes	Cottidae	*Myoxocephalus scorpius* (Linnaeus, 1758)	shorthorn sculpin	Magdalen Islands region of the Gulf of St. Lawrence	Montreuil 1958

**Table 2.** Definitive host records for *Corynosoma magdaleni*. All members of the family Phocidae, order Carnivora.

**Host order**	**Host family**	**Host species**	**Host vernacular name**	**Geographical locality**	**Reference**
Carnivora	Phocidae	*Halichoerus grypus* (Fabricius, 1791)	grey seal	Baltic Sea	Waindok et al. 2018
Carnivora	Phocidae	*Halichoerus grypus* (Fabricius, 1791)	grey seal	Baltic Sea, Baltic Proper, Bothnian Sea, Bothnian Bay, Sweden	Leidenberger and Bäcklin 2008
Carnivora	Phocidae	*Halichoerus grypus* (Fabricius, 1791)	grey seal	Baltic Sea, Finland, Gulf of Bothnia, Åland Island	Nickol et al. 2002
Carnivora	Phocidae	*Halichoerus grypus* (Fabricius, 1791)	grey seal	Baltic Sea, Sweden	Leidenberger et al. 2019
Carnivora	Phocidae	*Halichoerus grypus* (Fabricius, 1791)	grey seal	Eastern Canada	Montreuil 1958
Carnivora	Phocidae	*Phoca vitulina* Linnaeus, 1758	harbour seal	Eastern Canada	Montreuil 1958
Carnivora	Phocidae	*Phoca vitulina* Linnaeus, 1758	harbour seal	North Sea, Schleswig-Holstein	Waindok et al. 2018
Carnivora	Phocidae	*Phoca vitulina* Linnaeus, 1758 (as *Phoca vitulina concolor* De Kay, 1842)	harbour seal	Eastern Canada	Montreuil 1958
Carnivora	Phocidae	*Pusa hispida* (Schreber, 1775)	ringed seal	Baltic Sea	Leidenberger et al. 2019
Carnivora	Phocidae	*Pusa hispida* (Schreber, 1775)	ringed seal	Baltic Sea, Germany, Schleswig-Holstein	Waindok et al. 2018
Carnivora	Phocidae	*Pusa hispida* (Schreber, 1775)	ringed seal	Baltic Sea, Bothnian Bay	Valtonen et al. 2004
Carnivora	Phocidae	*Pusa hispida* (Schreber, 1775)	ringed seal	Baltic Sea, Gulf of Finland	Delyamure et al. 1980
Carnivora	Phocidae	*Pusa hispida* (Schreber, 1775)	ringed seal	European Arctic	Treshchev 1982* citet by Raga 1992
Carnivora	Phocidae	*Pusa hispida saimensis* Nordquist, 1899	Saimaa ringed seal	Lake Saimaa, Finland	Sinisalo et al. 2004, Aznar et al. 2006, García-Varela et al. 2011

**Table 3.** Definitive hosts of *Corynosoma semerme*. *Source not viewed, ^Found in Host-Parasite Database NHM London without further reference details.

**Host order**	**Host family**	**Host species**	**Host vernacular name**	**Geographical locality**	**Reference**
Artiodactyla	Monodontidae	*Delphinapterus leucas* (Pallas, 1776)	Beluga, Beluga whale, white whale	Bristol Bay, Alaska, Bering Sea	Neiland 1962, Measures et al. 1995
Carnivora	Odobenidae	*Odobenus rosmarus* (Linnaeus, 1758)	walrus	Arctic Ocean, Northern Atlantic	Delyamure 1961, von Sprehn 1966 cited by Raga 1992
Carnivora	Otariidae	*Callorhinus ursinus* (Linnaeus, 1758)	Northern fur seal	Alaska, Bering Sea, St. Paul Island	Kuzmina et al. 2012
Carnivora	Otariidae	*Callorhinus ursinus* (Linnaeus, 1758) (as *Callorhinus alascanus*)	Northern fur seal	North America	Van Cleave 1953
Carnivora	Phocidae	*Cystophora cristata* (Erxleben, 1777)	hooded seal	European Arctic	Delyamure 1961*, Treshchev 1982* cited by Raga 1992
Carnivora	Phocidae	*Erignathus barbatus* (Erxleben, 1777)	bearded seal	North America	Van Cleave 1953
Carnivora	Phocidae	*Erignathus barbatus* (Erxleben, 1777)	bearded seal	White Sea	Popova et al. 1975* cited by Raga 1992
Carnivora	Phocidae	*Halichoerus grypus* (Fabricius, 1791)	grey seal	Arctic Ocean, Northern Atlantic, European Arctic	von Sprehn 1966* cited by Raga 1992
Carnivora	Phocidae	*Halichoerus grypus* (Fabricius, 1791)	grey seal	Germany	Lühe 1911
Carnivora	Phocidae	*Halichoerus grypus* (Fabricius, 1791)	grey seal	Germany	Aznar et al. 2006
Carnivora	Phocidae	*Halichoerus grypus* (Fabricius, 1791)	grey seal	Baltic Sea, Finland, Gulf of Bothnia, Åland Island	Nickol et al. 2002
Carnivora	Phocidae	*Halichoerus grypus* (Fabricius, 1791)	grey seal	Baltic Sea, Baltic Proper, Bothnian Sea, Bothnian Bay, Sweden	Leidenberger and Bäcklin 2008
Carnivora	Phocidae	*Halichoerus grypus* (Fabricius, 1791)	grey seal	Baltic Sea, Germany, Schleswig-Holstein	Waindok et al. 2018
Carnivora	Phocidae	*Histriophoca fasciata* (Zimmerman, 1783) (as *Phoca fasciata*)	ribbon seal	Bering Sea, Alaska	Rausch et al. 1990
Carnivora	Phocidae	*Histriophoca fasciata* (Zimmerman, 1783) (as *Phoca fasciata*)	ribbon seal	Arctic Ocean, Bering Sea	Shults and Frost 1988
Carnivora	Phocidae	*Histriophoca fasciata* (Zimmerman, 1783) (as *Phoca fasciata*)	ribbon seal	Sea of Okhotsk	Shults and Frost 1988
Carnivora	Phocidae	*Pagophilus groenlandicus* (Erxleben, 1777) (as *Phoca groenlandica*)	harp seal, Greenland seal	Arctic Ocean, Northern Atlantic, European Arctic	von Sprehn 1966* cited by Raga 1992
Carnivora	Phocidae	*Phoca largha* Pallas, 1811	spotted seal, larga seal	Sea of Okhotsk	Sasaki et al. 2019
Carnivora	Phocidae	*Phoca largha* Pallas, 1811	spotted seal, larga seal	Russia	Aznar et al. 2006
Carnivora	Phocidae	*Phoca largha* Pallas, 1811	spotted seal, larga seal	Bering Sea, Alaska	Rausch et al. 1990
Carnivora	Phocidae	*Phoca largha* Pallas, 1811	spotted seal, larga seal	Arctic Ocean, Bering Sea	[On the helminthofauna of largi and harbour seals.]^
Carnivora	Phocidae	*Phoca largha* Pallas, 1811	spotted seal, larga seal	Sea of Okhotsk	[On the helminthofauna of largi and harbour seals.]^
Carnivora	Phocidae	*Phoca vitulina* Linnaeus, 1758	harbour seal	Germany	Lühe 1911
Carnivora	Phocidae	*Phoca vitulina* Linnaeus, 1758	harbour seal	Sweden	Lundström 1942
Carnivora	Phocidae	*Phoca vitulina* Linnaeus, 1758	harbour seal	North Sea, Germany, Schleswig-Holstein	Waindok et al. 2018
Carnivora	Phocidae	*Phoca vitulina* Linnaeus, 1758	harbour seal	Arctic Ocean, Northern Atlantic, European Arctic	von Sprehn 1966* cited by Raga 1992
Carnivora	Phocidae	*Phoca vitulina* Linnaeus, 1758	harbour seal	Alaska, Bering Sea, St. Paul Island	Kuzmina et al. 2012
Carnivora	Phocidae	*Pusa hispida* (Schreber, 1775)	ringed seal	Germany	Lühe 1911
Carnivora	Phocidae	*Pusa hispida* (Schreber, 1775)	ringed seal	Baltic Sea, Bothnian Bay, Finland	Helle and Valtonen 1981
Carnivora	Phocidae	*Pusa hispida* (Schreber, 1775)	ringed seal	Baltic Sea, Bothnian Bay, Finland	Valtonen and Helle 1988
Carnivora	Phocidae	*Pusa hispida* (Schreber, 1775)	ringed seal	Baltic Sea, Bothnian Bay, Finland	Valtonen et al. 2004
Carnivora	Phocidae	*Pusa hispida* (Schreber, 1775)	ringed seal	North America	Van Cleave 1953
Carnivora	Phocidae	*Pusa hispida* (Schreber, 1775) (as *Phoca foetida*)	ringed seal	Baltic Sea, Sweden, Östra Rönnskär	Forssell 1904
Carnivora	Phocidae	*Pusa hispida* (Schreber, 1775) (as *Phoca foetida*)	ringed seal	Baltic Sea, Finland, Tvärminne	Forssell 1905

**Table 4.** Paratenic hosts of *Corynosoma semerme*; all Actinopterygii. *Source not viewed.

**Host order**	**Host family**	**Host species**	**Host vernacular name**	**Geographical locality**	**Reference**
Acipenseriformes	Acipenseridae	*Acipenser sturio* Linnaeus, 1758	sturgeon	USSR (CIS)	Shulman 1954*
Acipenseriformes	Acipenseridae	*Acipenser sturio* Linnaeus, 1758	sturgeon	USSR (CIS)	Shulman 1954*
Anguilliformes	Anguillidae	*Anguilla anguilla* (Linnaeus, 1758)	European eel	Sweden	Lundström 1942
Anguilliformes	Anguillidae	*Anguilla anguilla* (Linnaeus, 1758)	European eel	Europe	Van Cleave 1953
Clupeiformes	Clupeidae	*Clupea harengus* Linnaeus, 1758	herring	Sweden	Lundström 1942
Clupeiformes	Clupeidae	*Clupea harengus* Linnaeus, 1758 (as *Clupea harengus membras*)	herring	Baltic Sea, Finland, Tvärminne	Forssell 1904
Clupeiformes	Clupeidae	*Clupea harengus* Linnaeus, 1758 (as *Clupea harengus membras*)	herring	Baltic Sea, Finland, Tvärminne	Forssell 1905
Clupeiformes	Clupeidae	*Clupea harengus* Linnaeus, 1758 (as *Clupea harengus membras*)	herring	Germany	Lühe 1911
Clupeiformes	Clupeidae	*Clupea harengus* Linnaeus, 1758 (as *Clupea harengus membras*)	herring	Europe	Van Cleave 1953
Cypriniformes	Cyprinidae	*Blicca bjoerkna* (Linnaeus, 1758)	white bream	Sweden	Lundström 1942
Cypriniformes	Cyprinidae	*Blicca bjoerkna* (Linnaeus, 1758)	white bream	Europe	Van Cleave 1953
Cypriniformes	Cyprinidae	*Pelecus cultratus* (Linnaeus, 1758)	ziege	Finno-Karelian ASSR	Rumyantsev and Ieshko 1997*
Esociformes	Esocidae	*Esox lucius* Linnaeus, 1758	pike	Baltic Sea, Finland, Tvärminne	Forssell 1905
Gadiformes	Gadidae	*Boreogadus saida* (Lepechin, 1774)	polar cod	Arctic Ocean, USSR zone, Pechora Sea	Karasev and Mitenev 1993*
Gadiformes	Gadidae	*Gadus morhua* Linnaeus, 1758	cod	Baltic Sea, Finland, Tvärminne	Forssell 1905
Gadiformes	Gadidae	*Gadus morhua* Linnaeus, 1758 (as *Gadus callarias*)	cod	Sweden	Lundström 1942
Gadiformes	Gadidae	*Gadus morhua* Linnaeus, 1758 (as *Gadus callarias*)	cod	Europe	Van Cleave 1953
Gadiformes	Lotidae	*Enchelyopus cimbrius* (Linnaeus, 1766) (as *Onos cimbrius*)	fourbeard rockling	Europe	Van Cleave 1953
Gadiformes	Lotidae	*Lota lota* (Linnaeus, 1758)	burbot	Baltic Sea, Bothnian Bay	Valtonen and Julkunen 1995
Gadiformes	Lotidae	*Lota lota* (Linnaeus, 1758) (as *Lota vulgaris*)	burbot	Sweden	Lundström 1942
Gadiformes	Lotidae	*Lota lota* (Linnaeus, 1758) (as *Lota vulgaris*)	burbot	Europe	Van Cleave 1953
Gadiformes	Lotidae	*Lota lota* (Linnaeus, 1758)	burbot	Tuluksak, Bering Sea, Alaska	Rausch et al. 1990
Gasterosteiformes	Gasterosteidae	*Gasterosteus aculeatus* Linnaeus, 1758	three-spined stickleback	Sweden	Lundström 1942
Lophiiformes	Lophiidae	*Lophius piscatorius* Linnaeus, 1758	angler	Sweden	Lundström 1942
Osmeriformes	Osmeridae	*Osmerus dentex* Steindachner and Kner, 1870	Pacific rainbow smelt	Japan	Sasaki et al. 2019
Osmeriformes	Osmeridae	*Osmerus eperlanus* (Linnaeus, 1758)	rainbow smelt	Baltic Sea, Finland, Tvärminne	Forssell 1904
Osmeriformes	Osmeridae	*Osmerus eperlanus* (Linnaeus, 1758)	rainbow smelt	Baltic Sea, Finland, Tvärminne	Forssell 1905
Osmeriformes	Osmeridae	*Osmerus eperlanus* (Linnaeus, 1758)	rainbow smelt	Germany	Lühe 1911
Osmeriformes	Osmeridae	*Osmerus eperlanus* (Linnaeus, 1758)	rainbow smelt	Sweden	Lundström 1942
Osmeriformes	Osmeridae	*Osmerus eperlanus* (Linnaeus, 1758)	rainbow smelt	Europe	Van Cleave 1953
Osmeriformes	Osmeridae	*Osmerus eperlanus* (Linnaeus, 1758)	rainbow smelt	Baltic Sea, Bothnian Bay	Valtonen and Julkunen 1995
Perciformes	Labridae	*Ctenolabrus rupestris* (Linnaeus, 1758)	Atlantic herring	southern Norway, Flødevigen,	Karlsbakk et al. 1996
Perciformes	Percidae	*Gymnocephalus cernua* (Linnaeus, 1758) (as *Acerina cernua*)	ruffe	Baltic Sea, Finland, Tvärminne	Forssell 1905
Perciformes	Percidae	*Gymnocephalus cernua* (Linnaeus, 1758) (as *Acerina cernua*)	ruffe	Germany	Lühe 1911
Perciformes	Percidae	*Gymnocephalus cernua* (Linnaeus, 1758) (as *Acerina cernua*)	ruffe	Sweden	Lundström 1942
Perciformes	Percidae	*Gymnocephalus cernua* (Linnaeus, 1758) (as *Acerina cernua*)	ruffe	Europe	Van Cleave 1953
Perciformes	Percidae	*Gymnocephalus cernua* (Linnaeus, 1758) (as *Gymnocephalus cernuus*)	ruffe	Baltic Sea, Bothnian Bay	Valtonen and Julkunen 1995
Perciformes	Percidae	*Perca fluviatilis* Linnaeus, 1758	perch	Baltic Sea, Finland, Tvärminne	Forssell 1905
Perciformes	Percidae	*Perca fluviatilis* Linnaeus, 1758	perch	Sweden	Lundström 1942
Perciformes	Percidae	*Perca fluviatilis* Linnaeus, 1758	perch	Europe	Van Cleave 1953
Perciformes	Percidae	*Sander lucioperca* (Linnaeus, 1758) (as *Stizostedion lucioperca*)	zanda, pike-perch	Finno-Karelian ASSR	Rumyantsev and Ieshko 1997*
Perciformes	Zoarcidae	*Lycodes raridens* Taranetz & Andriashev, 1937	marbled eelpout	Arctic Ocean, Bering Sea	Shults and Frost 1988
Perciformes	Zoarcidae	*Zoarces viviparus* (Linnaeus, 1758)	eelpout	Sweden	Lundström 1942
Perciformes	Zoarcidae	*Zoarces viviparus* (Linnaeus, 1758)	eelpout	Europe	Van Cleave 1953
Perciformes	Zoarcidae	*Zoarces viviparus* (Linnaeus, 1758)	eelpout	Baltic Sea, Estonia, Riga Bay	Vismanis et al. 1980*
Perciformes	Zoarcidae	*Zoarces viviparus* (Linnaeus, 1758)	eelpout	Baltic Sea, Germany, Lübecker Bay	Zander 1991
Pleuronectiformes	Pleuronectidae	*Hippoglossoides platessoides* (Fabricius, 1780) (as *Hippoglossoides platessoides limandoides)*	American plaice	Arctic Ocean, Greenland/Icelandic Zone, Icelandic coast	Olafsdottir 1999
Pleuronectiformes	Pleuronectidae	*Limanda limanda* (Linnaeus, 1758) (as *Pleuronectes limanda*)	common dab	Sweden	Lundström 1942
Pleuronectiformes	Pleuronectidae	*Limanda limanda* (Linnaeus, 1758) (as *Pleuronectes limanda*)	common dab	Europe	Van Cleave 1953
Pleuronectiformes	Pleuronectidae	*Platichthys flesus* (Linnaeus, 1758)	flounder	Baltic Sea, Finland, Tvärminne	Forssell 1905
Pleuronectiformes	Pleuronectidae	*Platichthys flesus* (Linnaeus, 1758)	flounder	Germany	Lühe 1911
Pleuronectiformes	Pleuronectidae	*Platichthys flesus* (Linnaeus, 1758)	flounder	Sweden	Lundström 1942
Pleuronectiformes	Pleuronectidae	*Platichthys flesus* (Linnaeus, 1758)	flounder	Sweden	Van Cleave 1953
Pleuronectiformes	Pleuronectidae	*Pleuronectes platessa* Linnaeus, 1758	European plaice	Europe	Van Cleave 1953
Pleuronectiformes	Pleuronectidae	*Pseudopleuronectes americanus* Walbaum, 1792	winter flounder	Atlantic, Canada	Arai 1989
Pleuronectiformes	Scophthalmidae	*Scophthalmus maximus* (Linnaeus, 1758) (as *Rhombus maximus*)	turbot	Baltic Sea, Finland, Tvärminne	Forssell 1904
Pleuronectiformes	Scophthalmidae	*Scophthalmus maximus* (Linnaeus, 1758) (as *Rhombus maximus*)	turbot	Baltic Sea, Finland, Tvärminne	Forssell 1905
Pleuronectiformes	Scophthalmidae	*Scophthalmus maximus* (Linnaeus, 1758) (as *Rhombus maximus*)	turbot	Germany	Lühe 1911
Pleuronectiformes	Scophthalmidae	*Scophthalmus maximus* (Linnaeus, 1758) (as *Rhombus maximus*)	turbot	Sweden	Lundström 1942
Pleuronectiformes	Scophthalmidae	*Scophthalmus maximus* (Linnaeus, 1758) (as *Rhombus maximus*)	turbot	Europe	Van Cleave 1953
Salmoniformes	Salmonidae	*Coregonus albula* (Linnaeus, 1758)	European cisco	Sweden	Lundström 1942
Salmoniformes	Salmonidae	*Coregonus albula* (Linnaeus, 1758)	European cisco	Europe	Van Cleave 1953
Salmoniformes	Salmonidae	*Coregonus fera* Jurine, 1825	true fera	Europe	Van Cleave 1953
Salmoniformes	Salmonidae	*Salmo salar* Linnaeus, 1758	salmon	European USSR (CIS)	Rumyantsev et al. 1998*
Salmoniformes	Salmonidae	*Salmo trutta* Linnaeus, 1758	brown trout, sea trout	European USSR (CIS)	Rumyantsev et al. 1998*
Salmoniformes	Salmonidae	*Thymallus thymallus* (Linnaeus, 1758)	grayling	Europe	Rumyantsev et al. 1999*
Scorpaeniformes	Cottidae	*Myoxocephalus quadricornis* (Linnaeus, 1758)	fourhorn sculpin	Baltic Sea, Bothnian Bay	Sinisalo and Valtonen 2003
Scorpaeniformes	Cottidae	*Myoxocephalus quadricornis* (Linnaeus, 1758) (as *Cottus quadricornis*)	fourhorn sculpin	Baltic Sea, Finland, Tvärminne	Forssell 1904
Scorpaeniformes	Cottidae	*Myoxocephalus quadricornis* (Linnaeus, 1758) (as *Cottus quadricornis*)	fourhorn sculpin	Baltic Sea, Finland, Tvärminne	Forssell 1905
Scorpaeniformes	Cottidae	*Myoxocephalus quadricornis* (Linnaeus, 1758) (as *Cottus quadricornis)*	fourhorn sculpin	Germany	Lühe 1911
Scorpaeniformes	Cottidae	*Myoxocephalus quadricornis* (Linnaeus, 1758) (as *Cottus quadricornis*)	fourhorn sculpin	Sweden	Lundström 1942
Scorpaeniformes	Cottidae	*Myoxocephalus quadricornis* (Linnaeus, 1758) (as *Cottus quadricornis*)	fourhorn sculpin	Europe	Van Cleave 1953
Scorpaeniformes	Cottidae	*Myoxocephalus scorpius* (Linnaeus, 1758)	shorthorn sculpin	Baltic Sea, Bothnian Bay	Valtonen and Julkunen 1995
Scorpaeniformes	Cottidae	*Myoxocephalus scorpius* (Linnaeus, 1758)	shorthorn sculpin	Baltic Sea, North-eastern Bothnian Bay	Valtonen et al. 2001
Scorpaeniformes	Cottidae	*Myoxocephalus scorpius* (Linnaeus, 1758) (as *Cottus scorpius*)	shorthorn sculpin	Sweden	Lundström 1942
Scorpaeniformes	Cottidae	*Myoxocephalus scorpius* (Linnaeus, 1758) *(as Cottus scorpius*)	shorthorn sculpin	Baltic Sea, Finland, Tvärminne	Forssell 1905
Scorpaeniformes	Cottidae	*Myoxocephalus scorpius* (Linnaeus, 1758) (as *Cottus scorpius*)	shorthorn sculpin	Europe	Van Cleave 1953
Scorpaeniformes	Cyclopteridae	*Cyclopterus lumpus* Linnaeus, 1758	goldsinny wrasse	Sweden	Lundström 1942
Scorpaeniformes	Cyclopteridae	*Cyclopterus lumpus* Linnaeus, 1758	goldsinny wrasse	Europe	Van Cleave 1953
Scorpaeniformes	Psychrolutidae	*Dasycottus setiger* Bean, 1890	spinyhead sculpin	Arctic Ocean, Bering Sea	Shults and Frost 1988

**Table 5.** Definitive hosts of *Corynosoma strumosum*. *Source not viewed. ^Found in Host-Parasite Database NHM London without further reference details.

**Host** **order**	**Host family**	**Host species**	**Host vernacular name**	**Geographical locality**	**Reference**
Artiodactyla	Monodontidae	*Delphinapterus leucas* (Pallas, 1776)	Beluga, Beluga whale, white whale	Bristol Bay, Alaska, Barent Sea, Sea of Okhotsk, North Pacific Ocean, Arctic	Krotov and Delyamure 1952*, Delyamure and Kleinenberg 1958*, Neiland 1962, Yablokov et al. 1972* all cited by Measures et al. 1995
Carnivora	Odobenidae	*Odobenus rosmarus* (Linnaeus, 1758)	walrus	no area specified	Dailey 1975, Raga 1992
Carnivora	Otariidae	*Callorhinus ursinus* (Linnaeus, 1758)	Northern fur seal	Alaska, Bering Sea, St. Paul Island	Ionita et al. 2008, Kuzmina et al. 2012
Carnivora	Otariidae	*Zalophus californianus* (Lesson, 1828)	California sea lion	California	Van Cleave 1953
Carnivora	Phocidae	*Cystophora cristata* (Erxleben, 1777)	hooded seal	Europe	Van Cleave 1953
Carnivora	Phocidae	*Erignathus barbatus* (Erxleben, 1777)	bearded seal	Alaska, Arctic	Van Cleave 1953
Carnivora	Phocidae	*Erignathus barbatus* (Erxleben, 1777)	bearded seal	Barent Sea, Sea of Okhotsk, Chukchi seas, Eastern Siberian Sea	Popov and Fortunato 1987
Carnivora	Phocidae	*Halichoerus grypus* (Fabricius, 1791)	grey seal	Germany	Lühe 1911
Carnivora	Phocidae	*Halichoerus grypus* (Fabricius, 1791)	grey seal	Holland, Germany	Aznar et al. 2006
Carnivora	Phocidae	*Halichoerus grypus* (Fabricius, 1791)	grey seal	Atlantic coast, Ireland	O'Neill and Whelan 2002
Carnivora	Phocidae	*Halichoerus grypus* (Fabricius, 1791)	grey seal	Baltic Sea, Finland, Gulf of Bothnia, Åland Island	Nickol et al. 2002
Carnivora	Phocidae	*Halichoerus grypus* (Fabricius, 1791)	grey seal	Baltic Sea, Baltic Proper, Bothnian Sea, Bothnian Bay, Sweden	Leidenberger and Bäcklin 2008
Carnivora	Phocidae	*Halichoerus grypus* (Fabricius, 1791)	grey seal	North Sea, Germany, Schleswig-Holstein	Waindok et al. 2018
Carnivora	Phocidae	*Halichoerus grypus* (Fabricius, 1791)	grey seal	Arctic Ocean, Northern Atlantic, European Arctic	Delyamure 1961*, von Sprehn 1966* cited by Raga 1992
Carnivora	Phocidae	*Halichoerus grypus* (Fabricius, 1791)	grey seal	Alaska, Arctic	Van Cleave 1953
Carnivora	Phocidae	*Histriophoca fasciata* (Zimmermann, 1783)	ribbon seal	Barent Sea, Sea of Okhotsk, Chukchi seas, Eastern Siberian Sea	Popov and Fortunato 1987
Carnivora	Phocidae	*Histriophoca fasciata* (Zimmermann, 1783) (as *Phoca fasciata*)	ribbon seal	Bering Sea, Alaska	Rausch et al. 1990
Carnivora	Phocidae	*Histriophoca fasciata* (Zimmermann, 1783) (as *Phoca fasciata*)	ribbon seal	Bering Sea	Shults and Frost 1988
Carnivora	Phocidae	*Pagophilus groenlandicus* (Erxleben, 1777)	harp seal	Greenland	Van Cleave 1953
Carnivora	Phocidae	*Pagophilus groenlandicus* (Erxleben, 1777) (as *Phoca groenlandica*)	harp seal	Germany	Lühe 1911
Carnivora	Phocidae	*Phoca largha* Pallas, 1811	spotted seal	Sea of Okhotsk, Japan Sea	Sasaki et al. 2019
Carnivora	Phocidae	*Phoca largha* Pallas, 1811	spotted seal	Bering Sea, Alaska	Rausch et al. 1990
Carnivora	Phocidae	*Phoca largha* Pallas, 1811	spotted seal	Bering Sea	[On the helminthofauna of largi and harbour seals.]^
Carnivora	Phocidae	*Phoca largha* Pallas, 1811	Larga seal, spotted seal	Barent Sea, Sea of Okhotsk, Chukchi seas, Eastern Siberian Sea	Popov and Fortunato 1987
Carnivora	Phocidae	*Phoca largha* Pallas, 1811	Larga seal, spotted seal	Russia: Anadyr Gulf	Aznar et al. 2006
Carnivora	Phocidae	*Phoca largha* Pallas, 1811	spotted seal	no area specified	[On the helminthofauna of largi and harbour seals.]^
Carnivora	Phocidae	*Phoca largha* Pallas, 1811	spotted seal	Japan, Hokkaido, Nemuro Peninsula	Nakaoka et al. 1986*
Carnivora	Phocidae	*Phoca vitulina* Linnaeus, 1758	harbour seal	Baltic Sea	Mühling 1989
Carnivora	Phocidae	*Phoca vitulina* Linnaeus, 1758	harbour seal	Germany	Rudolphi 1802
Carnivora	Phocidae	*Phoca vitulina* Linnaeus, 1758	harbour seal	Germany	Lühe 1911
Carnivora	Phocidae	*Phoca vitulina* Linnaeus, 1758	harbour seal	Sweden	Lundström 1942
Carnivora	Phocidae	*Phoca vitulina* Linnaeus, 1758	harbour seal	Baltic Sea and North Sea, Germany, Schleswig-Holstein	Waindok et al. 2018
Carnivora	Phocidae	*Phoca vitulina* Linnaeus, 1758	harbour seal	Alaska, Bering Sea, St. Paul Island	Kuzmina et al. 2012
Carnivora	Phocidae	*Phoca vitulina* Linnaeus, 1758	harbour seal	Alaska, Arctic	Van Cleave 1953
Carnivora	Phocidae	*Phoca vitulina* Linnaeus, 1758	harbour seal	USA, California, Monterey Bay	García-Varela et al. 2011
Carnivora	Phocidae	*Phoca vitulina* Linnaeus, 1758	harbour seal	Pacific	Sasaki et al. 2019
Carnivora	Phocidae	*Phoca vitulina* Linnaeus, 1758 (as *Phoca vitulina stejnegeri* J.A.Allen, 1902)	Kuril harbour seal	Japan, Hokkaido, Erimo Cape	Kaimoto et al. 2018
Carnivora	Phocidae	*Phoca vitulina* Linnaeus, 1758 (as *Phoca vitulina stejnegeri* J.A.Allen, 1902)	Kuril harbour seal	Japan, Hokkaido, Nemuro Peninsula	Nakaoka et al. 1986*
Carnivora	Phocidae	*Phoca vitulina richardii* (Gray, 1864)	Californian harbour seal	California	Van Cleave 1953
Carnivora	Phocidae	*Phoca vitulina richardii* (Gray, 1864)	Pacific harbor seal	Washington, Northern Pacific	Dailey and Fallace 1989
Carnivora	Phocidae	*Pusa caspica* (Gmelin, 1788)	Caspian seal	Iran, Mazandaran Province, Ramsar City	Amin et al. 2011
Carnivora	Phocidae	*Pusa caspica*, (Gmelin, 1788) (as *Phoca caspica* Gmelin, 1788)	Caspian seal	Caspian Sea	Kurochkin 1975* cited by Raga 1992
Carnivora	Phocidae	*Pusa hispida saimensis* (Nordquist, 1899)	Saimaa ringed seal	Lake Saimaa, Finland	Valtonen and Helle 1988
Carnivora	Phocidae	*Pusa hispida* (Schreber, 1775)	ringed seal	Germany	Lühe 1911
Carnivora	Phocidae	*Pusa hispida* (Schreber, 1775)	ringed seal	Sweden	Lundström 1942
Carnivora	Phocidae	*Pusa hispida* (Schreber, 1775)	ringed seal	Baltic Sea, Bothnian Bay, Finland	Helle and Valtonen 1981
Carnivora	Phocidae	*Pusa hispida* (Schreber, 1775)	ringed seal	Baltic Sea, Bothnian Bay, Finland	Valtonen and Helle 1988
Carnivora	Phocidae	*Pusa hispida* (Schreber, 1775)	ringed seal	Baltic Sea, Bothnian Bay, Finland	Valtonen et al. 2004
Carnivora	Phocidae	*Pusa hispida* (Schreber, 1775)	ringed seal	Baltic Sea, Sweden	Leidenberger et al. 2019
Carnivora	Phocidae	*Pusa hispida* (Schreber, 1775)	ringed seal	Alaska, Arctic	Van Cleave 1953
Carnivora	Phocidae	*Pusa hispida* (Schreber, 1775)	ringed seal	Barent Sea, Sea of Okhotsk, Chukchi seas, Eastern Siberian Sea	Popov and Fortunato 1987
Carnivora	Phocidae	*Pusa hispida* (Schreber, 1775) (as *Phoca foetida*)	ringed seal	Baltic Sea, Finland, Tvärminne	Forssell 1904
Carnivora	Phocidae	*Pusa hispida* (Schreber, 1775) (as *Phoca foetida*)	ringed seal	Baltic Sea, Finland, Tvärminne	Forssell 1905
Cetartiodactyla	Phocoeridae	*Phocoena phocoena* (Linnaeus, 1758)	harbour porpoise	Pacific	Sasaki et al. 2019
Cetartiodactyla	Phocoeridae	*Neophocaena phocaenoides* (G. Cuvier, 1829)	finless porpoise	Pacific	Sasaki et al. 2019

**Table 6.** Paratenic host records for *Corynosoma strumosum*. *Source not viewed, NA: not available, ^Found in Host-Parasite Database NHM London without further reference details.

**Host order**	**Host family**	**Host species**	**Host vernacular name**	**Geographical locality**	**Reference**
Acipenseriformes	Acipenseridae	*Acipenser gueldenstaedti* Brandt & Ratzeburg, 1833	Danube sturgeon	Caspian Sea, southern part, Sefid-Rud River	Sattari and Mokhayer 2005
Acipenseriformes	Acipenseridae	*Acipenser persicus* Borodin, 1897 (as *Acipenser gueldenstaedtipersicus natio kurensis*)	Persian sturgeon	Georgia, River Kura	Kurashvili et al. 1980*
Acipenseriformes	Acipenseridae	*Acipenser stellatus* Pallas, 1771	starry sturgeon	Caspian Sea, southern part, Sefid-Rud River	Sattari and Mokhayer 2005
Acipenseriformes	Acipenseridae	*Acipenser transmontanus* Richardson, 1836	white sturgeon	British Columbia, Canada	Arai 1989
Acipenseriformes	Acipenseridae	*Huso huso* (Linnaeus, 1758)	beluga	Caspian Sea, southern part, Sefid-Rud River	Sattari and Mokhayer 2005
Anguilliformes	Anguillidae	*Anguilla anguilla* (Linnaeus, 1758)	European eel	Germany	Lühe 1911
Anguilliformes	Congridae	*Conger conger* (Linnaeus, 1758)	European conger	Sweden	Lundström 1942
Anguilliformes	Congridae	*Conger conger* (Linnaeus, 1758)	European conger	Europe	Van Cleave 1953
Clupeiformes	Clupeidae	*Alosa braschnikowi* (Borodin, 1904) (as *Alosa brashnikovi grimmi*)	Caspian marine shad	Caspian Sea	Ibragimov and Vetchanin 1988*
Clupeiformes	Clupeidae	*Alosa braschnikowi* (Borodin, 1904) (as *Alosa brashnikovi kisselewitschi*)	Caspian marine shad	Caspian Sea	Ibragimov and Vetchanin 1988*
Clupeiformes	Clupeidae	*Alosa kessleri* (Grimm, 1887) (as *Alosa kessleri volgensis*)	Caspian anadromous shad	Caspian Sea	Izyumova 1977*
Clupeiformes	Clupeidae	*Alosa kessleri* (Grimm, 1887) (as *Alosa kessleri volgensis*)	Caspian anadromous shad	USSR (CIS)	Izyumova 1977*
Clupeiformes	Clupeidae	*Clupea harengus* Linnaeus, 1758	herring	Sweden	Lundström 1942
Clupeiformes	Clupeidae	*Clupea harengus* Linnaeus, 1758	herring	Europe	Van Cleave 1953
Clupeiformes	Clupeidae	*Clupea harengus* Linnaeus, 1758 (as *Clupea harengus membras*)	herring	Baltic Sea, Finland, Tvärminne	Forssell 1905
Clupeiformes	Clupeidae	*Clupea pallasii* Valenciennes, 1847	Pacific herring	Pacific ocean, Sea of Okhotsk	Sasaki et al. 2019
Clupeiformes	Clupeidae	*Clupea pallasii pallasii* Valenciennes, 1847 (as *Clupea harengus pallasii*)	Pacific herring	Pacific ocean, Canada	Arai 1989
Clupeiformes	Clupeidae	*Clupeonella caspia* Svetovidov, 1941 (as *Clupeonella delicatula caspia*)	Caspian tyulka	Caspian Sea	Ibragimov 1988*
Clupeiformes	Clupeidae	*Clupeonella cultriventris* (Nordmann, 1840) (as *Clupea cultriventris*)	Black Sea sprat	Caspian Sea	Shamsi et al. 1998*
Clupeiformes	Clupeidae	*Clupeonella grimmi* Kessler, 1877	Caspian sprat, big-eyed kilka	Capian Sea, southern part	Habibi and Shamsi 2018
Cypriniformes	Cyprinidae	*Abramis brama* (Linnaeus, 1758)	freshwater bream	Georgia, River Kura	Kurashvili et al. 1980*
Cypriniformes	Cyprinidae	*Alburnus chalcoides* (Güldenstädt, 1772) (as *Chalcalburnus chalcoides*)	shemaya	Georgia, River Kura (USSR)	Kurashvili et al. 1980*
Cypriniformes	Cyprinidae	*Alburnus chalcoides* (Güldenstädt, 1772) (as *Chalcalburnus chalcoides*)	shemaya	USSR (CIS)	Izyumova 1977*
Cypriniformes	Cyprinidae	*Barbus barbus* (Linnaeus, 1758)	barbel	USSR (CIS)	Izyumova 1977*
Cypriniformes	Cyprinidae	*Barbus brachycephalus caspius* Berg, 1914	Caspian barbel	River Kura (USSR)	Kurashvili et al. 1980*
Cypriniformes	Cyprinidae	*Carassius carassius* (Linnaeus, 1758)	Crucian carp	Azerbaijan	On the study of helminths of the crucian carp in Azerbaijan fish farms.
Cypriniformes	Cyprinidae	*Cyprinus carpio* Linnaeus, 1758	common carp	Georgia, River Kura (USSR)	Kurashvili et al. 1980*
Cypriniformes	Cyprinidae	*Leuciscus idus* (Linnaeus, 1758)	orfe, ide	Finno-Karelian ASSR	Rumyantsev and Ieshko 1997*
Cypriniformes	Cyprinidae	*Pelecus cultratus* (Linnaeus, 1758)	sabrefish, razorfish, knife	Finno-Karelian ASSR	Rumyantsev and Ieshko 1997*
Cypriniformes	Cyprinidae	*Rutilus frisii* (Nordmann, 1840) (as *Rutilus frisii kutum*)	Black Sea roach	Georgia, River Kura (USSR)	Kurashvili et al. 1980*
Cypriniformes	Cyprinidae	*Rutilus rutilus* (Linnaeus, 1758) (as *Rutilus rutilus caspicus natio knipowitschia*)	roach	Caspian Sea	Ibragimov 1989*
Cypriniformes	Cyprinidae	*Vimba vimba* (Linnaeus, 1758)	vimba bream	Lithuania	Rauckis 1988*
Esociformes	Esocidae	*Esox lucius* Linnaeus, 1758	pike	Baltic Sea, Finland, Tvärminne	Forssell 1905
Gadiformes	Gadidae	*Boreogadus saida* (Lepechin, 1774)	Polar cod	Arctic Ocean: USSR zone	Karasev and Mitenev 1993*
Gadiformes	Gadidae	*Eleginus gracilis* (Tilesius, 1810)	saffron cod	Gertner Bay, Cape of Nyuklya near Magadan	Skorobrechova and Nikishin 2014
Gadiformes	Gadidae	*Eleginus gracilis* (Tilesius, 1810)	saffron cod	Japan, Shikotan Island	Zhukov 1960*
Gadiformes	Gadidae	*Eleginus nawaga* (Walbaum, 1792) (as *Eleginus navaga*)	Arctic cod	Arctic Ocean: USSR zone	Karasev and Mitenev 1993*
Gadiformes	Gadidae	*Gadus chalcogrammus* Pallas, 1814 (as *Theragra chalcogramma*)	Alaska pollock	Bering Sea	Shults and Frost 1988
Gadiformes	Gadidae	*Gadus chalcogrammus* Pallas, 1814 (as *Theragra chalcogramma*)	Alaska pollock	Bering Sea	Avdeev and Avdeev 1998*
Gadiformes	Gadidae	*Gadus chalcogrammus* Pallas, 1814 (as *Theragra chalcogramma*)	Alaska pollock	Pacific ocean, Canada	Arai 1989
Gadiformes	Gadidae	*Gadus macrocephalus* Tilesius, 1810	Pacific cod	Pacific coast, USA and Canada	Van Cleave 1953
Gadiformes	Gadidae	*Gadus morhua* Linnaeus, 1758 (as *Gadus callarias*)	cod	Sweden	Lundström 1942
Gadiformes	Gadidae	*Gadus morhua* Linnaeus, 1758 (as *Gadus callarias*)	cod	Europe	Van Cleave 1953
Gadiformes	Gadidae	*Melanogrammus aeglefinus* (Linnaeus, 1758)	haddock	Atlantic, Canada	Arai 1989
Gadiformes	Lotidae	*Lota lota* (Linnaeus, 1758)	burbot	Baltic Sea, Bothnian Bay	Valtonen and Julkunen 1995
Gadiformes	Lotidae	*Lota lota* (Linnaeus, 1758) (as *Lota vulgaris*)	burbot	Baltic Sea, Finland, Tvärminne	Forssell 1905
Gadiformes	Lotidae	*Lota lota* (Linnaeus, 1758) (as *Lota vulgaris*)	burbot	Germany	Lühe 1911
Gadiformes	Lotidae	*Lota lota* (Linnaeus, 1758) (as *Lota vulgaris*)	burbot	Sweden	Lundström 1942
Gadiformes	Lotidae	*Lota lota* (Linnaeus, 1758) (as *Lota vulgaris*)	burbot	Europe	Van Cleave 1953
Gadiformes	Merlucciidae	*Macruronus novaezelandiae* (Hector, 1871)	whiptail, tailed hake, Patagonian whiphake, New Zealand whiptail, blue hake, blue grenadier	New Zealand coast	Klimpel et al. 2001*
Gadiformes	Merlucciidae	*Merluccius capensis* Castelnau, 1861	whiting, stockfish, South African whiting, shallow-water Cape hake, shallow water hake, hake, Cape hake	no area specified	Parukhin 1989*
Gasterosteiformes	Gasterosteidae	*Gasterosteus aculeatus* Linnaeus, 1758	three-spined stickleback	Sweden	Lundström 1942
Gasterosteiformes	Gasterosteidae	*Gasterosteus aculeatus* Linnaeus, 1758	three-spined stickleback	Europe	Van Cleave 1953
Gasterosteiformes	Gasterosteidae	*Gasterosteus aculeatus* Linnaeus, 1758	three-spined stickleback	Caspian Sea, southeast part, Gomishan Lagoon	Niksirat et al. 2006
Lophiiformes	Lophiidae	*Lophius piscatorius* Linnaeus, 1758	angler	Baltic Sea	Mühling 1989
Lophiiformes	Lophiidae	*Lophius piscatorius* Linnaeus, 1758	angler	Sweden	Lundström 1942
Lophiiformes	Lophiidae	*Lophius piscatorius* Linnaeus, 1758	angler	Europe	Van Cleave 1953
Lophiiformes	Lophiidae	*Lophius piscatorius* Linnaeus, 1758	monkfish, angler-fish	no area specified	Parukhin 1989*
Ophidiiformes	Ophidiidae	*Hoplobrotula gnathopus* (Regan, 1921)	false kinglip	no area specified	Parukhin 1989*
Osmeriformes	Osmeridae	*Hypomesus japonicus* (Brevoort, 1856)	Japanese smelt	Pacific ocean	Sasaki et al. 2019
Osmeriformes	Osmeridae	*Hypomesus japonicus* (Brevoort, 1856)	Japanese smelt	Japan, Shikotan Island	Zhukov 1960*
Osmeriformes	Osmeridae	*Hypomesus japonicus* (Brevoort, 1856)	Japanese smelt	Japan, Hokkaido, Erimo Cape	Araki and Machida 1987*
Osmeriformes	Osmeridae	*Hypomesus japonicus* (Brevoort, 1856) (as *Hypomesus preiosus japonicus*)	Japanese smelt	Japan, Hokkaido	Nagasawa et al. 1989*
Osmeriformes	Osmeridae	*Hypomesus olidus* (Pallas, 1814)	pond smelt	Nagaevo and Gertner Bay, Magadan, Russia, Sea of Okhotsk	Skorobrechova and Nikishin 2011
Osmeriformes	Osmeridae	*Hypomesus olidus* (Pallas, 1814)	pond smelt	Japan, Shikotan Island	Zhukov 1960*
Osmeriformes	Osmeridae	*Osmerus dentex* Steindachner and Kner, 1870	Pacific rainbow smelt	Japan, Sea of Okhotsk, Pacific ocean	Sasaki et al. 2019
Osmeriformes	Osmeridae	*Osmerus eperlanus* (Linnaeus, 1758)	rainbow smelt	Sweden	Lundström 1942
Osmeriformes	Osmeridae	*Osmerus eperlanus* (Linnaeus, 1758)	rainbow smelt	Europe	Van Cleave 1953
Osmeriformes	Osmeridae	*Osmerus eperlanus* (Linnaeus, 1758)	rainbow smelt	Baltic Sea, Bothnian Bay	Valtonen and Julkunen 1995
Osmeriformes	Osmeridae	*Osmerus mordax dentex* Steindachner & Kner, 1870	rainbow smelt	Nagaevo and Gertner Bay, Magadan, Russia, Sea of Okhotsk	Skorobrechova and Nikishin 2011
Osmeriformes	Osmeridae	*Spirinchus lanceolatus* (Hikita, 1913)	Sishamo smelt	Japan Hokkaido, Mukawa River	Fujita 1921*
Osmeriformes	Osmeridae	*Spirinchus lanceolatus* (Hikita, 1913) (as *Osmerus lanceolatus*)	Shishamo, willow leaf fish	Japan	Van Cleave 1953
Perciformes	Gobiidae	*Benthophilus stellatus* (Sauvage, 1874)	Stellate tadpole-goby	Caspian Sea	Ibragimov 1988*
Perciformes	Gobiidae	*Gobius* Linnaeus, 1758	goby	Caspian Sea	Ibragimov 1988*
Perciformes	Gobiidae	*Neogobius fluviatilis* (Pallas, 1814) (as *Gobius fluviatilis*)	monkey goby	Caspian Sea	Ibragimov 1988*
Perciformes	Gobiidae	*Neogobius fluviatilis* (Pallas, 1814) *(as Neogobius fluviatilis pallasi* (Pallas, 1814)	monkey goby	Caspian Sea, south-eastern part	Pazooki et al. 2011
Perciformes	Gobiidae	*Neogobius melanostomus* (Pallas, 1814)	round goby	Caspian Sea	Ibragimov 1988*
Perciformes	Gobiidae	*Ponticola bathybius* (Kessler, 1877) (as *Neogobius bathybius*)	NA	Capian Sea, south-eastern part	Pazooki et al. 2011
Perciformes	Gobiidae	*Ponticola gorlap* (Iljin, 1949) (as *Neogobius kesslerigorlap)*	Caspian bighead goby	Capian Sea, south-eastern part	Pazooki et al. 2011
Perciformes	Gobiidae	*Ponticola kessleri* (Günther, 1861) (as *Neogobius kessleri*)	bighead goby	Caspian Sea	Ibragimov 1988*
Perciformes	Hexagrammidae	*Hexagrammos octogrammus* (Pallas, 1814)	masked greenling	Sea of Okhotsk	Skorobrechova 2009* cited in Skorobrechova et al. 2011
Perciformes	Mugilidae	*Chelon auratus* (Risso, 1810) (as *Mugil auratus*)	golden grey mullet	Caspian Sea	Ibragimov 1988*
Perciformes	Mugilidae	*Chelon auratus* (Risso, 1810) (as *Mugil auratus*)	leaping mullet	Caspian Sea	Ibragimov 1988*
Perciformes	Percidae	*Gymnocephalus cernua* (Linnaeus, 1758) (as *Gymnocephalus cernuus*)	ruffe	Baltic Sea, Bothnian Bay	Valtonen and Julkunen 1995
Perciformes	Percidae	*Perca fluviatilis* Linnaeus, 1758	perch	Baltic Sea, Finland, Tvärminne	Forssell 1905
Perciformes	Percidae	*Perca fluviatilis* Linnaeus, 1758	perch	Germany	Lühe 1911
Perciformes	Percidae	*Perca fluviatilis* Linnaeus, 1758	perch	Europe	Van Cleave 1953
Perciformes	Percidae	*Sander lucioperca* (Linnaeus, 1758) (as *Lucioperca lucioperca*)	zander, pike-perch	Georgia, River Kura (USSR)	Kurashvili et al. 1980*
Perciformes	Percidae	*Sander lucioperca* (Linnaeus, 1758) (as *Lucioperca lucioperca*)	zander, pike-perch	USSR (CIS)	Izyumova 1977*
Perciformes	Percidae	*Sander lucioperca* (Linnaeus, 1758) (as *Stizostedion lucioperca*)	zander, pike-perch	Finno-Karelian ASSR	Rumyantsev and Ieshko 1997*
Perciformes	Sciaenidae	*Umbrina roncador* Jordan & Gilbert, 1882	yellowfin croaker	Catalina Island, California	Van Cleave 1953
Perciformes	Sparidae	*Pagrus pagrus* (Linnaeus, 1758)	red porgy, Couch's sea-bream, common seabream	no area specified	Parukhin 1966*
Perciformes	Sparidae	*Sparus heterodus* Peters, 1877	NA	no area specified	Parukhin 1966*
Perciformes	Sphyraenidae	*Sphyraena barracuda* (Edwards, 1771)	great barracuda	no area specified	Parukhin 1966*
Perciformes	Stichaeidae	*Pholidapus dybowskii* (Steindachner, 1880)	stichaeid fish	Japan, Shikotan Island	Zhukov 1960*
Perciformes	Trachinidae	*Trachinus draco* Linnaeus, 1758	weever	Baltic Sea	Mühling 1989
Perciformes	Trachinidae	*Trachinus draco* Linnaeus, 1758	weever	Sweden	Lundström 1942
Perciformes	Trachinidae	*Trachinus draco* Linnaeus, 1758	weever	Germany	Lühe 1911
Perciformes	Trachinidae	*Trachinus draco* Linnaeus, 1758	weever	Europe	Van Cleave 1953
Perciformes	Trichodontidae	*Arctoscopus japonicus* (Steindachner, 1881)	Japanese sandfish	Japan, Shikotan Island	Zhukov 1960*
Perciformes	Zoarcidae	*Lycodes raridens* Taranetz & Andriashev, 1937	marbled eelpout	Bering Sea	Shults and Frost 1988
Perciformes	Zoarcidae	*Hadropareia middendorffii* Schmidt, 1904	NA	Sea of Okhosk	Skorobrechova 2010* cited in Skorobrechova et al. 2011
Perciformes	Zoarcidae	*Zoarces viviparus* (Linnaeus, 1758)	eelpout	Baltic Sea, Polen, Gdansk Bight	Markowski 1938
Perciformes	Zoarcidae	*Zoarces viviparus* (Linnaeus, 1758)	eelpout	Sweden	Lundström 1942
Perciformes	Zoarcidae	*Zoarces viviparus* (Linnaeus, 1758)	eelpout	Europe	Van Cleave 1953
Perciformes	Zoarcidae	*Zoarces viviparus* (Linnaeus, 1758)	eelpout	Baltic Sea, Germany, Salzhaff	Reimer and Walter 1998
Pleuronectiformes	Pleuronectidae	*Atheresthes stomias* (Jordan & Gilbert, 1880)	arrow-tooth flounder	British Columbia	Wierzbicka and Piasecki 1998*
Pleuronectiformes	Pleuronectidae	*Eopsetta jordani* (Lockingston, 1879)	petrale sole	Canada	Arai 1989
Pleuronectiformes	Pleuronectidae	*Hippoglossoides platessoides* (Fabricius, 1780) (as *Hippoglossoides platessoides limandoides*)	sand-dab, long rough dab, Canadian plaice, American plaice	Arctic Ocean, Greenland/Icelandic zone	Olafsdottir 1999
Pleuronectiformes	Pleuronectidae	*Hippoglossus stenolepis* (Schmidt, 1904)	Pacific halibut	Northern Pacific	Blaylock et al. 1998*
Pleuronectiformes	Pleuronectidae	*Lepidopsetta bilineata* (Ayres, 1855)	rock sole	Pacific coast, USA and Canada	Van Cleave 1953
Pleuronectiformes	Pleuronectidae	*Lepidopsetta bilineata* (Ayres, 1855)	rock sole	Pacific ocean, Canada	Arai 1989
Pleuronectiformes	Pleuronectidae	*Limanda aspera* (Pallas, 1814)	yellowfin sole	Nagaevo and Gertner Bay, Magadan, Russia, Sea of Okhotsk	Skorobrechova and Nikishin 2011
Pleuronectiformes	Pleuronectidae	*Limanda limanda* (Linnaeus, 1758) (as *Pleuronectes limanda*)	common dab	Europe	Van Cleave 1953
Pleuronectiformes	Pleuronectidae	*Platichthys flesus* (Linnaeus, 1758)	flounder	Baltic Sea, Finland, Tvärminne	Forssell 1905
Pleuronectiformes	Pleuronectidae	*Platichthys flesus* (Linnaeus, 1758)	flounder	Germany	Lühe 1911
Pleuronectiformes	Pleuronectidae	*Platichthys flesus* (Linnaeus, 1758)	flounder	Sweden	Lundström 1942
Pleuronectiformes	Pleuronectidae	*Platichthys flesus* (Linnaeus, 1758)	perch	Sweden	Lundström 1942
Pleuronectiformes	Pleuronectidae	*Platichthys flesus* (Linnaeus, 1758) (as *Platessa flesus*)	flounder	Baltic Sea	Mühling 1989
Pleuronectiformes	Pleuronectidae	*Platichthys flesus* (Linnaeus, 1758) (as *Pleuronectes flesus*)	flounder	Baltic Sea, Finland, Tvärminne	Forssell 1905
Pleuronectiformes	Pleuronectidae	*Platichthys flesus* (Linnaeus, 1758) (as *Pleuronectes flesus*)	flounder	Europe	Van Cleave 1953
Pleuronectiformes	Pleuronectidae	*Platichthys stellatus* (Pallas, 1787)	starry flounder	Japan, Shikotan Island	Zhukov 1960*
Pleuronectiformes	Pleuronectidae	*Platichthys stellatus* (Pallas, 1787)	starry flounder	Pacific coast, USA and Canada	Van Cleave 1953
Pleuronectiformes	Pleuronectidae	*Platichthys stellatus* (Pallas, 1787)	starry flounder	Pacific ocean, Canada	Arai 1989
Pleuronectiformes	Pleuronectidae	*Pleuronectes obscurus* Herzenstein, 1890	black plaice	Japan, Shikotan Island	Zhukov 1960*
Pleuronectiformes	Pleuronectidae	*Reinhardtius hippoglossoides* (Walbaum, 1792)	turbot	Atlantic, Canada	Arai 1989
Pleuronectiformes	Pleuronectidae	*Verasper moseri* Jordan & Gilbert, 1898	barfin flounder	Japan, Hokkaido, Erimo Cape	Araki and Machida 1987*
Pleuronectiformes	Scophthalmidae	*Scophthalmus maximus* (Linnaeus, 1758) (as *Rhombus maximus*)	trubot	Baltic Sea, Finland, Tvärminne	Forssell 1904
Pleuronectiformes	Scophthalmidae	*Scophthalmus maximus* (Linnaeus, 1758) (as *Rhombus maximus*)	trubot	Baltic Sea, Finland, Tvärminne	Forssell 1905
Pleuronectiformes	Scophthalmidae	*Scophthalmus maximus* (Linnaeus, 1758) (as *Rhombus maximus*)	trubot	Germany	Lühe 1911
Pleuronectiformes	Scophthalmidae	*Scophthalmus maximus* (Linnaeus, 1758) (as *Rhombus maximus*)	trubot	Sweden	Lundström 1942
Pleuronectiformes	Scophthalmidae	*Scophthalmus maximus* (Linnaeus, 1758) (as *Rhombus maximus*)	trubot	Europe	Van Cleave 1953
Salmoniformes	Hemitripteridae	*Hemitripterus villosus* (Pallas, 1814)	shaggy sea raven	Japan, Hokkaido, Nemuro Peninsula	Nakaoka et al. 1986*
Salmoniformes	Salmonidae	*Coregonus fera* Jurine, 1825	true fera	Europe	Van Cleave 1953
Salmoniformes	Salmonidae	*Coregonus lavaretus* (Linnaeus, 1758)	lavaret	Europe	Van Cleave 1953
Salmoniformes	Salmonidae	*Oncorhynchus gorbuscha* (Walbaum, 1792)	pink salmon	Pacific ocean, Canada	Arai 1989
Salmoniformes	Salmonidae	*Oncorhynchus gorbuscha* (Walbaum, 1792)	pink salmon	Sakhalin Island	Vyalova 2003*
Salmoniformes	Salmonidae	*Oncorhynchus keta* (Walbaum, 1792)	chum salmon	Sakhalin Island	Vyalova 2003*
Salmoniformes	Salmonidae	*Oncorhynchus masou* (Brevoort, 1856) (as *Oncorhynchus masu*)	masu salmon	Russia	Ermolenko et al. 1989*
Salmoniformes	Salmonidae	*Oncorhynchus nerka* (Walbaum, 1792)	sockeye salmon	Pacific ocean, Canada	Arai 1989
Salmoniformes	Salmonidae	*Salmo salar* Linnaeus, 1758	salmon	European USSR (CIS)	Rumyantsev et al. 1998*
Salmoniformes	Salmonidae	*Salmo trutta* Linnaeus, 1758	trout	European USSR (CIS)	Rumyantsev et al. 1998*
Salmoniformes	Salmonidae	*Salvelinus alpinus alpinus* (Linnaeus, 1758)	Arctic charr	Canada, Quebec, Ungava Bay	Desdevises et al. 1998*
Salmoniformes	Salmonidae	*Salvelinus alpinus alpinus* (Linnaeus, 1758) (as *Salvelinus alpinus*)	Arctic charr	River Chaun	[On the helminthofauna of fish of the River Chaun.]^
Salmoniformes	Salmonidae	*Salvelinus leucomaenis leucomaenis* (Pallas, 1814)	white-spotted charr	Japan, Shikotan Island	Zhukov 1960*
Salmoniformes	Salmonidae	*Salvelinus leucomaenis leucomaenis* (Pallas, 1814) (as *Salvelinus leucomaenis*)	white-spotted charr	Japan, Hokkaido	Nagasawa et al. 1989*
Salmoniformes	Salmonidae	*Salvelinus malma* (Walbaum, 1792)	dolly varden	Russia	Ermolenko 1994*
Salmoniformes	Salmonidae	*Salvelinus malma* (Walbaum, 1792)	dolly varden	Russia	Ermolenko et al. 1989*
Salmoniformes	Salmonidae	*Thymallus thymallus* (Linnaeus, 1758)	grayling	European waters of NE Atlantic and Mediterranean	Rumyantsev et al. 1999*
Scorpaeniformes	Cottidae	*Gymnocanthus galeatus* Bean, 1881	armoured sculpin	Bering Sea	Shults and Frost 1988
Scorpaeniformes	Cottidae	*Gymnocanthus herzensteini* Jordan & Starks, 1904	staghorn sculpin	Japan, Hokkaido, Erimo Cape	Araki and Machida 1987*
Scorpaeniformes	Cottidae	*Leptocottus armatus* Girard, 1854	Pacific staghorn sculpin	Pacific coast, USA and Canada	Van Cleave 1953
Scorpaeniformes	Cottidae	*Myoxocephalus brandtii* (Steindachner, 1867)	snowy sculpin	Japan, Shikotan Island	Zhukov 1960*
Scorpaeniformes	Cottidae	*Myoxocephalus polyacanthocephalus* (Pallas, 1814) (as *Ainocottus ensiger*)	great sculpin	Japan, Hokkaido, Erimo Cape	Araki and Machida 1987*
Scorpaeniformes	Cottidae	*Myoxocephalus quadricornis* (Linnaeus, 1758)	fourhorn sculpin	Baltic Sea, Bothnian Bay	Sinisalo and Valtonen 2003
Scorpaeniformes	Cottidae	*Myoxocephalus quadricornis* (Linnaeus, 1758) (as *Cottus quadricornis*)	fourhorn sculpin	Sweden	Lundström 1942
Scorpaeniformes	Cottidae	*Myoxocephalus quadricornis* (Linnaeus, 1758) (as *Cottus quadricornis*)	fourhorn sculpin	Baltic Sea, Finland, Tvärminne	Forssell 1904
Scorpaeniformes	Cottidae	*Myoxocephalus quadricornis* (Linnaeus, 1758) (as *Cottus quadricornis*)	fourhorn sculpin	Baltic Sea, Finland, Tvärminne	Forssell 1905
Scorpaeniformes	Cottidae	*Myoxocephalus quadricornis* (Linnaeus, 1758) (as *Cottus quadricornis*)	fourhorn sculpin	Germany	Lühe 1911
Scorpaeniformes	Cottidae	*Myoxocephalus quadricornis* (Linnaeus, 1758) (as *Cottus quadricornis*)	fourhorn sculpin	Sweden	Lundström 1942
Scorpaeniformes	Cottidae	*Myoxocephalus quadricornis* (Linnaeus, 1758) (as *Cottus quadricornis*)	fourhorn sculpin	Europe	Van Cleave 1953
Scorpaeniformes	Cottidae	*Myoxocephalus scorpius* (Linnaeus, 1758)	fourhorn sculpin	Baltic Sea, Bothnian Bay	Valtonen and Julkunen 1995
Scorpaeniformes	Cottidae	*Myoxocephalus scorpius* (Linnaeus, 1758)	fourhorn sculpin	Baltic Sea, North-eastern Bothnian Bay	Valtonen et al. 2001
Scorpaeniformes	Cottidae	*Myoxocephalus scorpius* (Linnaeus, 1758) (as *Cottus scorpius*)	shorthorn sculpin	Germany	Lühe 1911
Scorpaeniformes	Cottidae	*Myoxocephalus scorpius* (Linnaeus, 1758) (as *Cottus scorpius*)	shorthorn sculpin	Sweden	Lundström 1942
Scorpaeniformes	Cottidae	*Myoxocephalus scorpius* (Linnaeus, 1758) (as *Cottus scorpius*)	fourhorn sculpin	Europe	Van Cleave 1953
Scorpaeniformes	Cottidae	*Myoxocephalus stelleri* (Tilesius, 1811)	Steller's sculpin	Gertner Bay, Cape of Nyuklya near Magadan	Skorobrechova and Nikishin 2014
Scorpaeniformes	Cottidae	*Myoxocephalus stelleri* (Tilesius, 1811)	Steller's sculpin	Sea of Okhotsk	Sasaki et al. 2019
Scorpaeniformes	Cottidae	*Taurulus bubalis* (Euphrasen, 1786)	sea scorpion	Germany	Lühe 1911
Scorpaeniformes	Cyclopteridae	*Cyclopterus lumpus* (Linnaeus, 1758)	lumpfish	Baltic Sea	Mühling 1989
Scorpaeniformes	Cyclopteridae	*Cyclopterus lumpus* (Linnaeus, 1758)	lumpfish	Germany	Lühe 1911
Scorpaeniformes	Cyclopteridae	*Cyclopterus lumpus* (Linnaeus, 1758)	lumpfish	Sweden	Lundström 1942
Scorpaeniformes	Cyclopteridae	*Cyclopterus lumpus* (Linnaeus, 1758)	lumpfish	Europe	Van Cleave 1953
Scorpaeniformes	Hemitripteridae	*Hemitripterus villosus* (Pallas, 1814)	shaggy sea raven	Japan, Shikotan Island	Zhukov 1960*
Scorpaeniformes	Hexagrammidae	*Hexagrammos lagocephalus* (Pallas, 1810)	rock greenling	Japan, Shikotan Island	Zhukov 1960*
Scorpaeniformes	Hexagrammidae	*Hexagrammos lagocephalus* (Pallas, 1810)	rock greenling	Japan, Hokkaido, Nemuro Peninsula	Nakaoka et al. 1986*
Scorpaeniformes	Hexagrammidae	*Hexagrammos octogrammus* (Pallas, 1814)	masked greenling	Japan, Shikotan Island	Zhukov 1960*
Scorpaeniformes	Hexagrammidae	*Hexagrammos stelleri* Tilesius, 1810	whitespotted greenling	Gertner Bay, Cape of Nyuklya near Magadan	Skorobrechova and Nikishin 2014
Scorpaeniformes	Hexagrammidae	*Pleurogrammus azonus* Jordan and Metz, 1913	Okhotsk atka mackerel	Sea of Okhotsk	Sasaki et al. 2019
Scorpaeniformes	Sebastidae	*Sebastes mentella* Travin, 1951	deepwater redfish, beaked redfish	NW Atlantic: Norwegian Sea and Barents Sea	Bakay 2001
Scorpaeniformes	Sebastidae	*Sebastes paucispinis* Ayres, 1854	bocaccio	Pacific coast of USA	Love et al. 2002*
Scorpaeniformes	Sebastidae	*Sebastes trivittatus* Hilgendorf, 1880	threestripe rockfish	Japan, Shikotan Island	Zhukov 1960*
Scorpaeniformes	Sebastidae	*Sebastes trivittatus* Hilgendorf, 1880	threestripe rockfish	Pacific ocean	Sasaki et al. 2019
Scorpaeniformes	Triglidae	*Chelidonichthys capensis* (Cuvier, 1829) (as *Trigla capensis*)	Cape gurnard	no area specified	Parukhin 1966*
Syngnathiformes	Syngnathidae	*Syngnathus* sp. Linnaeus, 1758	seaweed pipefishes	Caspian Sea	Ibragimov 1988*
Pleuronectiformes	Pleuronectidae	*Pseudopleuronectes herzensteini* (Jordan and Snyder, 1901) (as *Pleuronectes herzensteini*)	yellow striped flounder	Pacific ocean	Sasaki et al. 2019
Pleuronectiformes	Pleuronectidae	*Pseudopleuronectes obscurus* Herzenstein, 1890 (as *Pleuronectes obscurus*)	darkflounder	Pacific ocean	Sasaki et al. 2019
Petromyzontiformes	Petromyzontidae	*Caspiomyzon wagneri* (Kessler, 1870) (as *Caspiomyzon wagneri caspius*)	Caspian lamprey	Georgia, River Kura (USSR)	Kurashvili et al. 1980*
Petromyzontiformes	Petromyzontidae	*Lampetra fluviatilis* (Linnaeus, 1758)	river lamprey	Germany	Lühe 1911
Petromyzontiformes	Petromyzontidae	*Lampetra fluviatilis* (Linnaeus, 1758) (as *Petromyzon fluviatilis*)	river lamprey	Baltic Sea	Mühling 1989

**Table 7.** Accidental hosts of *Corynosoma semerme*. *Source not viewed, ^Found in Host-Parasite Database NHM London without further reference details.

**Host order**	**Host family**	**Host species**	**Host vernacular name**	**Geographical locality**	**Reference**
Anseriformes	Anatidae	*Clangula hyemalis* (Linnaeus, 1758) (as *Nyroca hyemalis*)	long-tailed duck	Germany	Lühe 1911
Charadriiformes	Laridae	*Larus fuscus* Linnaeus, 1758	black-backed gull	Baltic Sea, Finland	Forssell 1905
Suliformes	Phalacrocoracidae	*Phalacrocorax carbo* (Linnaeus, 1758)	great cormorant	Baltic Sea, Finland	Forssell 1905
Suliformes	Phalacrocoracidae	*Phalacrocorax carbo* (Linnaeus, 1758)	great cormorant	Germany	Lühe 1911
Suliformes	Phalacrocoracidae	*Phalacrocorax pelagicus* Pallas, 1811 (as *Phalacrocorax pelagicus pelagicus*)	pelagic cormorant	North America	Van Cleave 1953
Carnivora	Canidae	*Canis lupus familiaris* Linnaeus, 1758 (as *Canis familiaris*)	Husky dog	North America	Van Cleave 1953
Carnivora	Canidae	*Vulpes lagopus* Linnaeus, 1758 (as *Alopex lagopus*)	Arctic fox, polar fox	European USSR (CIS)	Yushkov 1995*
Carnivora	Canidae	*Vulpes lagopus* Linnaeus, 1758 (as *Alopex lagopus*)	Arctic fox, polar fox	St. Lawrence Island, Bering Sea, Alaska	Rausch et al. 1990
Carnivora	Mustelidae	*Mustela erminea* Linnaeus, 1758	stoat	Sweden	Lundström 1942
Carnivora	Mustelidae	*Mustela putorius* Linnaeus, 1758 (as *Putorius foetorius*)	European polecat	Germany	Lühe 1911
Carnivora	Mustelidae	*Neovison vison* (Schreber, 1777) (as *Mustela vison*)	mink	Finland	Nuorteva 1966
Carnivora	Mustelidae	*Neovison vison* (Schreber, 1777) (as *Mustela vison*)	American Mink	Iceland	Skirnisson 1995
Cetartiodactyla	Eschrichtiidae	*Eschrichtius robustus* (Lilljeborg, 1861) (as *Eschrichtius gibbosus*)	gray/grey whale	Arctic Ocean, Pacific Ocean	[Whales and dolphins. Monographic outline.]^
Cetartiodactyla	Phocoenidae	*Phocoena phocoena* (Linnaeus, 1758)	harbour porpoise	Germany	Lühe 1911
Cetartiodactyla	Phocoenidae	*Phocoena phocoena* (Linnaeus, 1758)	harbour porpoise	Northern Atlantic	[Whales and dolphins. Monographic outline.]^
Cetartiodactyla	Phocoenidae	*Phocoena phocoena* (Linnaeus, 1758)	harbour porpoise	Arctic Ocean, Northern Pacific Ocean	[Whales and dolphins. Monographic outline.]^
Cetartiodactyla	Phocoenidae	*Phocoena phocoena* (Linnaeus, 1758) (as *Phocoena communis*)	harbour porpoise	Finland, Tvärminne	Forssell 1904
Cetartiodactyla	Phocoenidae	*Phocoena phocoena* (Linnaeus, 1758) (as *Phocoena communis*)	harbour porpoise	Baltic Sea, Finland	Forssell 1905

**Table 8.** Accidental hosts of *Corynosoma strumosum.* * Source not viewed.

**Host order**	**Host family**	**Host species**	**Host vernacular name**	**Graphical locality**	**Reference**
Accipitriformes	Accipitridae	*Haliaeetus leucocephalus* (Linnaeus, 1766)	Bald eagle	North America, Alaska	Van Cleave 1953
Anseriformes	Anatidae	*Clangula hyemalis* (Linnaeus, 1758)	oldsquaw	Europe	Mühling 1989
Anseriformes	Anatidae	*Mergus merganser* Linnaeus, 1758	common merganser	Germany	Lühe 1911
Anseriformes	Anatidae	*Mergus serrator* Linnaeus, 1758	red-breasted merganser	Germany	Lühe 1911
Anseriformes	Anatidae	*Somateria mollissima* (Linnaeus, 1758)	eider duck	Iceland	Skirnisson and Jonsson 1996
Charadriiformes	Alcidae	*Uria lomvia* (Linnaeus, 1758)	thick-billed murre	Point Barrow, Alaska	Rausch et al. 1990
Charadriiformes	Laridae	*Larus argentatus* Pontoppidan, 1763	herring gull	Finland	Forssell 1905
Charadriiformes	Laridae	*Larus glaucescens* Naumann, 1840	glaucoused-winged gull	Napaskiak, Alaska	Rausch et al. 1990
Charadriiformes	Laridae	*Larus hyperboreus* Gunnerus, 1767	glaucous gull	Bear Island, Barents Sea	Sagerup et al. 2000
Charadriiformes	Laridae	*Sterna hirundo* Linnaeus, 1758	common tern	Sweden	Lundström 1942
Podicipediformes	Podicipedidae	*Podiceps grisegena* (Boddaert, 1783)	red-necked grebe	Germany	Lühe 1911
Suliformes	Phalacrocoracidae	*Phalacrocorax auritus* (Lesson, 1831)	double-crested cormorant	Germany	Lühe 1911
Suliformes	Phalacrocoracidae	*Phalacrocorax carbo* (Linnaeus, 1758)	great cormorant	France: Bretagne, Baltic Sea: Finland and Germany	Forssell 1905, Lühe 1911, Reimer 2002*
Suliformes	Phalacrocoracidae	*Phalacrocorax pelagicus* Pallas, 1811	pelagic cormorant	Barents Sea, Alaska	Rausch et al. 1990
Carnivora	Canidae	*Canis familiaris* Linnaeus, 1758	domestic dog	Alaska	Van Cleave 1953
Carnivora	Canidae	*Canis familiaris* Linnaeus, 1758	domestic dog (as sledge dog)	Tununak, Alaska	Rausch et al. 1990
Carnivora	Canidae	*Vulpes lagopus* (Linnaeus, 1758) (as *Alopex lagopus*)	Arctic fox, polar fox	St. Lawrence Island, Bering Sea, Alaska	Rausch et al. 1990
Carnivora	Canidae	*Vulpes vulpes* (Llinnaeus, 1758)	red fox	Hopper Bay, Cold Bay, Alaska	Rausch et al. 1990
Carnivora	Felidae	*Felis catus* Linnaeus, 1758	domestic cat	Germany	Lühe 1911
Carnivora	Mustelidae	*Enhydra lutris* (Linnaeus, 1758)	sea otter	Alaska	Van Cleave 1953
Carnivora	Mustelidae	*Lutra lutra* (Linnaeus, 1758)	European otter	Eire, Northern Ireland, England, Wales, Isle of Man, Orkneys, Shetlands, Scotland	McCarthy and Hassett 1993*, Jefferies et al. 1990, Weber 1991
Carnivora	Mustelidae	*Mustela putorius* Linnaeus, 1758	European polecat	Germany	Lühe 1911
Carnivora	Mustelidae	*Neovison vison* (Schreber, 1777)	mink	Oregon, North America	Van Cleave 1953
Carnivora	Mustelidae	*Neovison vison* (Schreber, 1777)	mink	Finland	Nuorteva 1966
Cetacea	Phocoenidae	*Phocoena phocoena* (Linnaeus, 1758)	harbour porpoise	Tvärminne, Finland	Forssell 1904, Forssell 1905, Lühe 1911
Primates	Hominidae	*Homo sapiens* Linnaeus, 1758	human	Chevak, Alaska	Rausch et al. 1990
